# Spatial event cluster detection using an approximate normal distribution

**DOI:** 10.1186/1476-072X-7-61

**Published:** 2008-12-12

**Authors:** Mahmoud Torabi, Rhonda J Rosychuk

**Affiliations:** 1 Department of Pediatrics, University of Alberta, Edmonton, Alberta, Canada

## Abstract

**Background:**

In geographic surveillance of disease, areas with large numbers of disease cases are to be identified so that investigations of the causes of high disease rates can be pursued. Areas with high rates are called disease clusters and statistical cluster detection tests are used to identify geographic areas with higher disease rates than expected by chance alone. Typically cluster detection tests are applied to incident or prevalent cases of disease, but surveillance of disease-related events, where an individual may have multiple events, may also be of interest. Previously, a compound Poisson approach that detects clusters of events by testing individual areas that may be combined with their neighbours has been proposed. However, the relevant probabilities from the compound Poisson distribution are obtained from a recursion relation that can be cumbersome if the number of events are large or analyses by strata are performed. We propose a simpler approach that uses an approximate normal distribution. This method is very easy to implement and is applicable to situations where the population sizes are large and the population distribution by important strata may differ by area. We demonstrate the approach on pediatric self-inflicted injury presentations to emergency departments and compare the results for probabilities based on the recursion and the normal approach. We also implement a Monte Carlo simulation to study the performance of the proposed approach.

**Results:**

In a self-inflicted injury data example, the normal approach identifies twelve out of thirteen of the same clusters as the compound Poisson approach, noting that the compound Poisson method detects twelve significant clusters in total. Through simulation studies, the normal approach well approximates the compound Poisson approach for a variety of different population sizes and case and event thresholds.

**Conclusion:**

A drawback of the compound Poisson approach is that the relevant probabilities must be determined through a recursion relation and such calculations can be computationally intensive if the cluster size is relatively large or if analyses are conducted with strata variables. On the other hand, the normal approach is very flexible, easily implemented, and hence, more appealing for users. Moreover, the concepts may be more easily conveyed to non-statisticians interested in understanding the methodology associated with cluster detection test results.

## Background

A cluster is an aggregation of excess cases of a disease or illness. When the aggregation occurs in geography, geographic cluster detection methods can be used to identify geographic areas that have higher numbers of cases than would be expected by chance. Statistical methods are used to identify such aggregations and once areas are identified, resources can be targeted to determine if a statistically significant cluster is spurious or uncover reasons for the cluster (e.g., environmental contamination).

In some contexts the number of cases may not be the sole focus of the most meaningful unit for cluster detection. For studies of health services utilization, the patient may access various sources of health care numerous times. A particular feature of these data are that an individual can provide multiple, correlated data points. Higher utilization may be indicative of more serious disease. Such services can be generally called disease-related events and detection of geographic areas with excess disease-related events is a different exercise than the identification of excess incident or prevalent cases.

Statistical cluster detection methods are generally classified into two main categories: non-focused (also often referred to as general) and focused. Non-focused tests of clustering identify areas with excess numbers of cases whereas focused tests identify areas with excess numbers of cases in the vicinity of potential causes (e.g., toxic waste site). There are several methods that are applicable in different situations. Some methods assume similar population sizes in each geographic area. Our interest, and application, focuses on methods that allow for diverse population sizes among the geographic areas.

Overviews of cluster detection methods can be found in Lawson et al. [1] and Waller and Gotway [2]. An exploratory approach involving many overlapping circles was introduced in Openshaw et al. [3]. Turnbull et al. [4] create overlapping circles with constant disease risk that partition the study region. While Turnbull et al. used a Monte Carlo simulation to assess statistical significance, Kulldorff and Nagarwalla [5] provided a generalization that uses a likelihood ratio test. Using a similar likelihood ratio test, Duczmal and Assunção [6] examine connected subgraphs. A chi-square statistic that compares observed to expected frequencies proposed by Tango [7] gives a test of clustering based on a "closeness measure" that provides an overall determination of the tendency to cluster in a study region.

Besag and Newell [8] take a different approach. They propose a test for each area based on the number of neighbours that must be combined in order to contain a minimum number of cases (i.e., cluster size). This method relies on a pre-determined cluster size for each test and Le et al. [9] provide a testing algorithm for the automatic selection of cluster sizes. More recently, Rosychuk et al. [10] proposed an event cluster detection method that was similar in spirit to the method of Besag and Newell. Rather than combining administrative areas in order to achieve a particular number of cases, areas are combined in order to contain at least a certain number of disease-related events. The probability of observing the number of events is based on a compound Poisson distribution and the relevant probabilities are obtained through a recursion relation. To our knowledge, this is the only method that allows for multiple, correlated events per case in its calculations.

In this paper, we propose the use of an approximate normal to the compound Poisson distribution. This approximation facilitates easier implementation and is appropriate for situations where the population sizes are large and the population distribution by important strata may differ by area. We begin by describing the event cluster detection method and the normal approach. We use a testing algorithm analogous to those proposed in Le et al. [8] and Rosychuk et al. [10]. We illustrate both approaches and compare the results for a data set on repeated visits to emergency departments. The performance of the proposed approach is also studied through the Monte Carlo simulation.

## Results

Our main result is the development and implementation of an approximate normal for the detection of geographic clusters of multiple (correlated) events. To help fix ideas, we first provide notation consistent with what appears in [10]. We assume that a study region is divided into separate, non-overlapping, administrative areas called cells and that each of these cells has a representative middle point called a centroid. Such a centroid could be geography- or population-based. We use the pairwise distance between cell centroids as a criteria to calculate the spatial relationship between cells. The total number of cells in the study region is denoted by *I*. We label cell *i*_*p *_as the *p*-*th *closest cell to cell *i*, *p *∈ {1,..., *I *- 1}, and *i*_0 _= *i*. Let *N*_*i *_be the population size of the *i*-*th *cell with the total population $N=\sum_{i=1}^{I}N_{i}$. A case is defined as an individual person with at least one disease-related event. Let *C*_*i *_and *C*_*ix *_be the number of cases and number of cases with exactly *x *events in the *i*-*th *cell, respectively, with observed values of *c*_*i *_and *c*_*ix*_. We also have *C*_*i *_= Σ_*x *_*C*_*ix*_, and the random variable *V*_*i *_= Σ_*x *_*xC*_*ix *_denotes the number of events in cell *i *with the observed value of *v*_*i*_. Assuming that *C*_*i *_and *V*_*i *_are finite, *C *= Σ_*i *_*C*_*i *_and *V *= Σ_*i *_*V*_*i *_denote the total number of cases and events for the entire region, respectively, with observed values of *c *and *v*.

The test is based on combining neighbouring cells to achieve a number of events above a threshold. The null hypothesis is that every individual is equally likely to have events, independent of other individuals and the location of residence. In contrast, the alternative hypothesis suggests that the number of events is higher than expected by the event distribution.

We first review the compound Poisson method described in Rosychuk et al., RHP hereafter, for event clustering in next section and we then introduce the new approach for general testing of events based on a normal distribution.

### Compound Poisson RHP approach

Each cell is tested separately. An event cluster size, *k*, is chosen that represents the minimum size of a cluster to be detected. For cell *i*, the test statistic is defined as the minimum number of cells that need to be combined with cell *i *to include the nearest *k *events,

(1) $$L_{i}=\min\left\{q:k\leq \sum_{p=0}^{q}V_{i_{p}}\right\}.$$

For cell *i*, let $N_{i:l}=\sum_{p=0}^{l}N_{i_{p}}$ be the total population in its *l *nearest neighbours. Then, under the null hypothesis, the number of cases in its *l *nearest neighbours, $C_{i:l}=\sum_{p=0}^{l}C_{i_{p}}$, follows a Poisson distribution with mean *λ*_*i*:*l *_= *N*_*i*:*l*_*C*/*N*. Since each case has at least one event and potentially many events, the significance level of each cell is determined by assuming that the number of events in combined cells follows a compound Poisson distribution [11]. That is, the number of events in combined cells can be considered as a random sum of a random number of events. We then have $V_{i:l}=\sum_{p=0}^{l}V_{i_{p}}=\sum_{j=1}^{C_{i:l}}{\text{Y}}_{j}$ as the total number of events for the *N*_*i*:*l *_individuals where *Y*_*j *_is a random variable and denotes the number of events of the *j*-*th *case within cell *i *and its *l *nearest neighbours, *j *= 1,..., *C*_*i*:*l*_. Hence, *V*_*i*:*l *_has a compound Poisson distribution under the null hypothesis. We may write the significance level as

(2) $$Pr(L_{i}\leq l)=1-\sum_{z=0}^{k-1}P_{i:l}(z),$$

where *P*_*i*:*l*_(*z*) = *Pr*(*V*_*i*:*l *_= *z*). Note that the probability *P*_*i*:*l*_(*z*) may be obtained through a recursion relation [11] where

(3) $$\begin{array}{c} P_{i:l}(0)=e^{-\lambda_{i:l}},\\ \begin{array}{cc} P_{i:l}(z)=\frac{\lambda_{i:l}}{z}\sum_{x=1}^{z}xQ(x)P_{i:l}(z-x) & z\geq 1, \end{array} \end{array}$$

and *Q*(*x*) = *P*(*Y*_*j *_= *x*). RHP used estimates ${\hat{\lambda }}_{i:l}$ = *N*_*i*:*l*_*c*/*N *and $\hat{Q}$(*x*) = *c*_+*x*_/*c *in where *c*_+*x *_is the observed number of cases in the entire region with exactly *x *events.

To get the cluster size *k*, RHP adopted the approach suggested by Le et al. which chooses multiple cluster sizes for each cell that depend on the population. RHP extended their approach to include strata information and also used a Monte Carlo simulation to evaluate overall clustering in the region.

### Approximate normal distribution method

The recursion relation can be cumbersome when we have a large number of events in each region. This computation is more difficult when we have strata with auxiliary information. We propose the normal distribution to approximate the compound Poisson probabilities. This approach well approximates the compound Poisson distribution when the mean of a cell is large enough [12,13]. Our approach follows the same idea as the method proposed by RHP, however, we use the normal distribution to calculate the significance level in each cell rather than using a compound Poisson distribution.

Let *C *denote the number of cases in the entire region and suppose *C *is a Poisson random variable with mean *λ*. Let *X*_1_,..., *X*_*C *_be independent random variables with identical distribution *F *with mean *μ *and variance *σ*^2^. Then $S=\sum_{i=1}^{C}X_{i}$ is a compound Poisson random variable with mean *λμ *and variance *λ*(*μ*^2 ^+ *σ*^2^). For large *λ*, the distribution of the compound Poisson can be approximated by a normal distribution with mean *λμ *and variance *λ*(*μ*^2 ^+ *σ*^2^). The normal distribution and compound Poisson distribution have the same mean and variance, however, it is easier to calculate quantiles from the normal distribution than from the compound Poisson distribution.

More precisely, the test statistic in our approach is based on the number of cells required to be combined to include at least k* events and is given by

(4) $$L_{i}^{*}=\min\left\{q:k^{*}\leq \sum_{p=0}^{q}V_{i_{p}}\right\}.$$

Our test statistic is different from the test statistic of RHP in the sense that $V_{i_{p}}$ in (4) has different distribution than $V_{i_{p}}$ in (1). The significance level of each cell is determined by assuming that the number of events in combined cells follows an approximate normal distribution. As in RHP, $V_{i:l}=\sum_{p=0}^{l}V_{i_{p}}=\sum_{j=1}^{C_{i:l}}{\text{Y}}_{j}$ is the total number of events for the *N*_*i*:*l *_individuals. Under the null hypothesis, the number of events of the *j*-*th *case, Y_*j*_, is equal to Y for each case and *Pr*(*Y *= *x*) is the same for all cells. Under the null hypothesis, $V_{i:l}=\sum_{j=1}^{C_{i:l}}{\text{Y}}_{j}$ has a normal distribution with mean *μ*_*i*:*l *_≡ *λ*_*i*:*l*_*E*(*Y*) and variance $\sigma_{i:l}^{2}\equiv \lambda_{i:l}E({\text{Y}}^{2})$. Assuming *Pr*(*Y*_*j *_= *x*) = *Q*(*x*), we may write $E(\text{Y})=\sum_{x=1}^{\infty }xPr(\text{Y}=x)=\sum_{x=1}^{\infty }xQ(x)$ and $E({\text{Y}}^{2})=\sum_{x=1}^{\infty }x^{2}Pr(\text{Y}=x)=\sum_{x=1}^{\infty }x^{2}Q(x)$. Therefore, the significance level becomes

(5) $$Pr(L_{i}^{*}\leq 1)≃1-\Phi \left(\frac{k^{*}-0.5-\mu_{i:l}}{\sigma_{i:l}}\right)+\Phi \left(\frac{-0.5-\mu_{i:l}}{\sigma_{i:l}}\right),$$

where Φ(·) is the cumulative distribution of the standard normal.

In practice, we use estimates ${\hat{\mu }}_{i:l}={\hat{\lambda }}_{i:l}\hat{E(\text{Y})}$ and ${\hat{\sigma }}_{i:l}^{2}={\hat{\lambda }}_{i:l}\hat{E({\text{Y}}^{2})}$ where

$$\hat{E(\text{Y})}=\sum_{x=1}^{\infty }x\hat{Q}(x)=\frac{1}{c}\sum_{x=1}^{\infty }xc_{+x}=\frac{1}{c}\sum_{i}\sum_{x}xc_{ix}=v/c,$$

and

$$\hat{E({\text{Y}}^{2})}=\sum_{x=1}^{\infty }x^{2}\hat{Q}(x)=\frac{1}{c}\sum_{x=1}^{\infty }x^{2}c_{+x}=\frac{1}{c}\sum_{i}\sum_{x}x^{2}c_{ix}=v^{*}/c,$$

with $v_{i}^{*}=\sum_{x}x^{2}c_{ix}$ and $v^{*}=\sum_{i}v_{i}^{*}$. For the regularity conditions on the mean and variance of the conditional distribution of the number of events for each case, the value of v*, and consequently *v*, *c *and *x*, must be bounded. Therefore, we have ${\hat{\mu }}_{i:l}$=*N*_*i:l*_*v*/*N* and ${\hat{\sigma }}_{i:l}^{2}$ = *N*_*i*:*l*_v*/*N*, noting that ${\hat{\lambda }}_{i:l}$ = *N*_*i*:*l*_*c*/*N *assuming *N *is finite. As a result, using (5) to find the significance level for each cell is more convenient than the compound Poisson distribution proposed by RHP. We have provided R [14] code available at  for potential users.

To better ascertain the chance of identified clusters, the observed number of significant cells at level *α *can be compared with the expectation under the null hypothesis to provide an overall assessment of statistical significance. A Monte Carlo simulation study can be used for this assessment by generating a large number of samples (simulated data sets), performing the test on each sample, and determining the proportion of samples that exceed the observed number of significant cells at level *α*. The samples are generated by conditioning on the *c*_1_, *c*_2_,..., cases. For each event number *x*, the *c*_*ix *_cases are randomly assigned to the cells based on the population size of each cell.

A similar approach can be taken for each cell *i *(= 1,..., *I*) to determine a Monte Carlo *p*-value, $p_{i}^{\left(MC\right)}$. Using the same simulated data sets as above, the proportion of simulations for cell *i *with a *p*-value at least as extreme as the *p*-value from the actual data is used as the Monte Carlo *p*-value for cell *i*. This Monte Carlo *p*-value is used as a criterion of how likely cell *i *is part of a cluster under the null hypothesis.

The event cluster size can be chosen and different situations may dictate particular choices for the cluster size. In absence of a meaningful size known *a priori*, an automatic testing algorithm can be used. We follow the approach used by Le et al. [8] and RHP to test cell *i *at a few different cluster sizes $k_{i0}^{*}$, $k_{i1}^{*}$, and $k_{i2}^{*}$ in sequence. In our adaptation, these cluster sizes are chosen based on normal distribution for the population in cell *i *alone, and with its nearest first and second neighbours. We consider $k_{i0}^{*}$ - 1, $k_{i1}^{*}$ - 1, and $k_{i2}^{*}$ - 1 to be the 95 percentile of normal distribution with means *μ*_*i*:0_, *μ*_*i*:1_, and *μ*_*i*:2_, and variances $\sigma_{i:0}^{2}$, $\sigma_{i:1}^{2}$, and $\sigma_{i:2}^{2}$, respectively. The cell *i *is tested at cluster size $k_{i0}^{*}$. It is only tested at sizes $k_{i1}^{*}$ and $k_{i2}^{*}$ if the tests based on $k_{i0}^{*}$ and $k_{i1}^{*}$, respectively, are insignificant. Essentially, a sequence of tests is conducted until either statistical significance is obtained or the last test in the sequence is completed. Because the normal distribution could have a non-integer 95 percentile, all cluster sizes were rounded up to the nearest integer. We note that this testing algorithm should provide the minimum number of events necessary for significance to be guaranteed based on the populations of cell *i *and its two nearest neighbours for sufficiently large *μ*_*i*_/*σ*_*i*_. For small *μ*_*i*_/*σ*_*i*_, the k* chosen using the 95th percentile of the normal distribution with mean *μ*_*i *_and variance $\sigma_{i}^{2}$ is smaller than the the minimum k* necessary to have (5) be less than 0.05. In that situation, the k* should be chosen to be the minimum integer required to have (5) be less than 0.05. If the cluster sizes are arbitrarily chosen, a too small choice would mean that a statistically significant result may not be possible and a too large choice would mean that a real cluster might not be identified because the combined cells might dilute the high rate.

We can also easily modify the approach to present a stratified cluster detection method. Let *C*_*s *_and *N*_*s *_be the total number of cases and population in stratum *s *(= 1,..., *S*) in the entire region, respectively. Let the number of cases and population of stratum *s *on its *l *nearest neighbours from cell *i *be *C*_*is*:*l *_and *N*_*is*:*l*_, respectively. Therefore, the *C*_*is*:*l *_follows a Poisson distribution with mean *λ*_*is*:*l *_= *N*_*is*:*l*_*C*_*s*_/*N*_*s*_. In addition, $V_{i:l}=\sum_{s=1}^{S}\sum_{j=1}^{C_{is:l}}{\text{Y}}_{js}$ is the total number of events for the *N*_*i*:*l *_case, where Y_*js *_is the number of events of the *j*-*th *case in stratum *s*, *j *= 1,..., *C*_*is*:*l *_with probabilities *Q*_*s*_(*x*) = *Pr*(*Y*_*js *_= *x*) for all *j *and events *x *≥ 1. Thus, the *V*_*i*:*l *_has a normal distribution with mean $\mu_{i:l}\equiv \sum_{s=1}^{S}\mu_{is:l}=\sum_{s=1}^{S}\lambda_{is:l}E({\text{Y}}_{js})$ and variance $\sigma_{i:l}^{2}\equiv \sum_{s=1}^{S}\sigma_{is:l}^{2}=\sum_{s=1}^{S}\lambda_{is:l}E({\text{Y}}_{js}^{2})$ where $E({\text{Y}}_{js})=\sum_{x=1}^{\infty }xPr({\text{Y}}_{js}=x)=\sum_{x=1}^{\infty }xQ_{s}(x)$ and $E({\text{Y}}_{js}^{2})=\sum_{x=1}^{\infty }x^{2}Pr({\text{Y}}_{js}=x)=\sum_{x=1}^{\infty }x^{2}Q_{s}(x)$. Note that *Q*_*s*_(*x*) can be estimated by *c*_+*sx*_/*c*_++*s*_, where *c*_+*sx *_is the number of cases with exactly *x *events in stratum *s *and *c*_++*s *_is the observed value of *C*_++*s*_, the total number of cases in stratum *s*. Thus, we can get the significance test similar to (5). The Monte Carlo simulation and cluster size choices proceed in a similar manner.

### Self-inflicted injury presentations to emergency departments

The western Canadian province of Alberta is divided into nine regional health authorities (HAs), with varying geographic and population sizes. In terms of administrative responsibility, these nine HAs are further sub-divided into (*I *= 68) subregional HAs and these subregional HAs are the geographic unit used in our analysis. We use the same emergency department (ED) data illustrated in RHP.

The Ambulatory Care Classification System (ACCS) captures a variety of outpatient services and includes all emergency department encounters in Alberta. This system was created in 1997 and provides a rich source of information on the health services required by the province's population. We focus on the pediatric population (less than 18 years of age) and ED presentations for self-inflicted injuries (SIIs). In our application, an individual with at least one SII presentation to an Alberta ED during the fiscal year 1998/99 is considered to be a case. These cases would be indicative of individuals suffering from self-harming behaviours. Since a case can make multiple presentations for SIIs during the study period, each SII presentation can be considered an event. Cases with high numbers of events may represent individuals with greater illness or with lesser access to other health resources. All of the data and pairwise distances between HAs were provided by Alberta Health and Wellness.

The population of the entire region under study was 785,079 and 827 children (cases) had 915 events (ED presentations for SII) during the 1998/1999 fiscal year. Analyses used gender and year of age as strata. The majority of cases had only one event, however, 54 cases had between two and eighteen SIIs during the study period.

**Figure 1:**
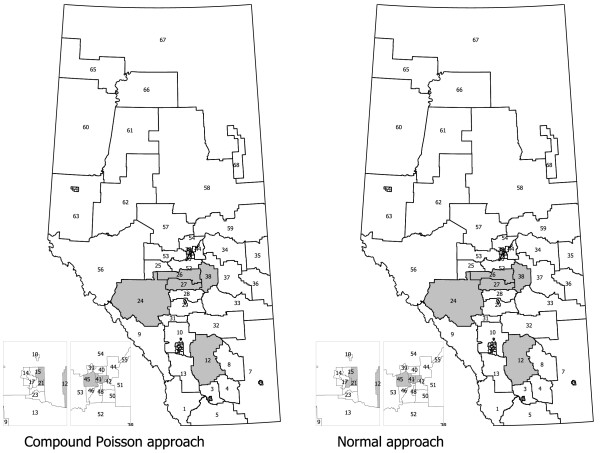
Subregional health authorities (HAs) identified as clusters on their own or when combined with neighbours to form clusters for the compound Poisson and normal approaches.

The results of the compound Poisson approach proposed by RHP and our approach are reported in Additional file 1 and the cells identified as parts of clusters are provided in Figure 1. The Additional file 1 shows the results of all of the tests conducted using the sequential testing algorithm. The results are very similar in the sense that the cluster sizes *k*_*i *_and $k_{i}^{*}$, test statistics *l*_*i *_and $l_{i}^{*}$, number of observed visits *v*_*i*:*l*_, and the population sizes *N*_*i*:*l*_, for each combined region are identical or nearly identical with each approach. Based on our approach, thirteen HAs were identified as clusters and of these seven HAs identified as clusters alone, three with the first nearest neighbour combined, and three with the two nearest neighbours combined. Table 1 displays the *p*-values for statistically significant clusters.

**Table 1:** The *p*-values for each significant HA for RHP method ($p_{i}^{CP}$), normal approach ($p_{i}^{N}$) and Monte Carlo ($p_{i}^{MC(CP)}$) and ($p_{i}^{MC(N)}$) for RHP and normal methods, respectively.

*i*	$p_{i}^{CP}$	$p_{i}^{N}$	$p_{i}^{MC(CP)}$	$p_{i}^{MC(N)}$
2	0.044*	0.035*	0.019*	0.018*
12	0.047*	0.035*	0.063	0.007*
16	0.047*	0.044*	0.035*	0.041*
20	0.968	0.043*	0.950	0.055
21	0.048*	0.028*	0.063	0.000*
24	0.046*	0.037*	0.066	0.006*
26	0.044*	0.026*	0.012*	0.000*
27	0.043*	0.040*	0.030*	0.018*
29	0.044*	0.033*	0.040*	0.000*
38	0.044*	0.033*	0.051	0.002*
41	0.045*	0.028*	0.039*	0.000*
42	0.038*	0.033*	0.000*	0.004*
45	0.043*	0.038*	0.011*	0.028*

An asterisk (*) denotes significant clusters at the 5% level, unadjusted for multiple testing.

The method of RHP based on the compound Poisson distribution distinguishes twelve HAs as clusters. All of these 12 HAs are also clusters identified in our method. For each of these HAs, the testing algorithm stops at the same point and the test statistics are identical. The cluster sizes are generally quite close, usually within one or two of each other. The observed events meet or exceed either cluster size and hence, both tests yield significant tests. For example, HA2 requires at least 30 events to be considered a cluster by itself under the compound Poisson and our approaches. With 33 observed events, both tests are significant. For HA12 to be a significant cluster on its own, the cluster sizes are 15 and 16 under the compound Poisson and normal methods, respectively (not shown). With only 12 events, the observed events are less than the cluster sizes and neither test is significant. When a nearest neighbour (HA21) is considered, the cluster sizes become 38 under both methods and with 42 observed events, both cluster sizes are less than 42 observed events and both tests are significant.

The only differences between the two approaches is HA20. HA20 is detected as cluster in our method, however, it is not identified as cluster in RHP method. As shown in Additional file 1, HA20 is identified as cluster in our method when combined with its two nearest neighbours. For the RHP approach, the sequence of cluster sizes tested are 21, 29, and 51 and the values for our approach are 22, 30, and 50. With 6 events in HA20 alone, the number of events is not as large as the cluster size of 21 and 22 in both approaches, and then the algorithm tests the next cluster size. In next step, the number of events combined with its first nearest neighbour (HA19) is 12 which is less than the cluster size 29 and 30 for both methods. With an insignificant result, the algorithm moves to the next cluster size in the sequence to test. HA20 is combined with its two nearest neighbours (HA19 and HA21) to yield a total of 50 events. With 50 events and a cluster size 50 in our approach, the test is significant. However, 50 events is less than the cluster size of 51 in the RHP approach and the test is not significant. In this situation, a slightly larger cluster size provided a different finding.

As noted earlier, the majority of clusters were individual HAs (i.e., *l *= 0). For cells 2, 21, 24, 26, 29, 41, and 42, both approaches yield significant clusters without combining any neighbours. When combined with cell 21, cells 12 and 16 are significant clusters in both methods. Cell 27 is a significant cluster in both approaches when combined with cells 26 and 28. When combined with cell 26, cell 38 is a significant cluster in both methods. Cell 45 is also a significant cluster in both approaches when combined with cells 41 and 47.

In general, the testing algorithm proposed by Le et al. and modified for event data is applied to choose a cluster size for an HA and to detect an individual cluster provided there are sufficient events. If an HA forms a cluster on its own, no further testing assesses if when combined with some of its nearest neighbours, the combined area is also a significant cluster. However, an individual HA may not be a cluster itself because of insufficient events to reach the significance level based on normal distribution. Combining the population of neighbours may produce enough events to detect a cluster with two or more (at most three based on our assumption) HAs.

In order to check the likely number of clusters detected, we implemented a Monte Carlo simulation with 1000 samples. No samples provided more than 12 clusters. Thus, these detected clusters are not likely all spurious and an overall *p*-value is calculated as 0. We also conducted Monte Carlo *p*-values, from the normal *p*-values, for each cell and observed that 12 of the significant HAs have $p_{i}^{MC(N)}$ less than 0.05 (Table 1). The Monte Carlo *p*-value for HA20 is 0.055 and is larger than corresponding normal *p*-value. The Monte Carlo *p*-values for the compound Poisson method $p_{i}^{MC(CP)}$ are also shown in Table 1 for the compound Poisson method. Using a custom R [14] program on an Windows PC platform, the normal approach takes 130 seconds to complete the individual and overall tests. For the compound Poisson approach, an existing C/C++ program on a Linux platform required 970 seconds to perform the calculations.

### Simulation study

We investigate the performance of the normal approach through a Monte Carlo simulation study. We set *I *= 50 as the number of cells in a 10 × 5 grid pattern. We based our experiment on nine different scenarios, where one cell (*i *= 28) has a higher rate than the other cells. We consider three different population sizes for each cell and three different settings for the total number of cases and events, (*c*, *v*), for each population size. We examine the ability of the approaches to identify a "true" cluster comprised of one cell (*i *= 28), by setting the rate in that cell to be 1.5 times larger than the rate in every other cell,

*p*_*i *_= *r *(*i *= 1,..., *I*; *i *∉ 28) and *p*_28 _= 1.5*r*,

where *r *is obtained based on the condition $\sum_{i=1}^{I}p_{i}=1$. To allow for each cell to have a different mean and variance, each cell's population size was determined randomly based on a mean population setting (e.g., if the mean population size was set to 5000, the population size of each cell was a random whole number in (4500,5500)). Note that the cases are generated in each simulation based on multinomial distribution where mean and variance for cell *i *are *N*_*i*_*p*_*i *_and *N*_*i*_*p*_*i*_(1 - *p*_*i*_), respectively. We implement a Monte Carlo simulation with 1000 samples per scenario. We summarize the nine different scenarios in Table 2.

**Table 2:** The nine different scenarios for the simulation study based on the mean population size, the number of cases, the number of events, and the number of cases for each event number *x *(*c*_+1_, *c*_+2_, *c*_+3_, *c*_+4_).

Scenario	Mean population size	(*c*, *v*)/50	(*c*_+1_, *c*_+2_, *c*_+3_, *c*_+4_)/50
1	5000	(5,11)	(2,1,1,1)
2	5000	(5,12)	(1,2,1,1)
3	5000	(5,14)	(1,1,1,2)

4	10000	(10,22)	(4,2,2,2)
5	10000	(10,24)	(2,4,2,2)
6	10000	(10,28)	(2,2,2,4)

7	20000	(20,44)	(8,4,4,4)
8	20000	(20,48)	(4,8,4,4)
9	20000	(20,56)	(4,4,4,8)

The samples are generated by conditioning on the {*c*_*x*_} cases. For each event number *x*, the *c*_*x *_cases are randomly assigned (multinomial distribution) to the cells based on the population size of each cell. In scenario 1, for example, the mean population size per cell is 5000 and the total number of cases and events are 5 × 50 = 250 and 11 × 50 = 550, respectively. As it is probably easier to think of the number of cases and events in a particular cell, the settings involve multiplications by the number of cells (50) to help interpretation. In each of the 1000 simulations, 100 cases have one event each at these 100 cases are randomly assigned to the 50 cells using a multinomial distribution with probability $\frac{1.5}{50.5}$ = 0.0297 for cell 28 and probability $\frac{1}{50.5}$ = 0.0198 for all other cells. Similarly, a total of 50 cases associated with two events each are randomly assigned to the 50 cells using a multinomial distribution with the probability of 0.0297 and 0.0198, for cell 28 and all other cells, respectively. This process continues until all cases have been randomly assigned to cells.

Note that we are not generating data based on a compound Poisson distribution. Rather, we are distributing cases amongst the cells according to a multinomial distribution as was done for the Monte Carlo simulations to assess overall clustering and in a similar manner to the Besag and Newell approach for cases. These simulations assess the ability of the compound Poisson and normal approaches to identify the "true" cluster. In essence, both the compound Poisson and normal approaches will be an approximation to the true distribution. While we have different numbers of cases and events, (*c*_*i*_, *v*_*i*_), for different cells and samples, the total number of cases and events, (*c*, *v*), are the same for each sample within a scenario. The generation of the data preserves the total number of cases and events.

**Figure 2:**
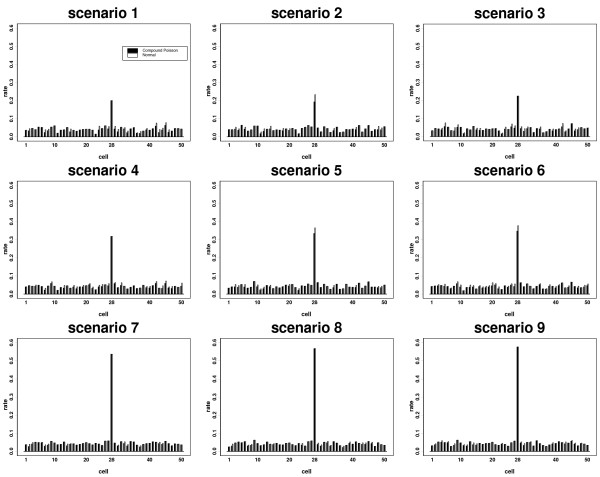
The rate of significant clusters based on 1000 Monte Carlo simulation for scenarios 1–9.

We identified the significant cluster based on the cell itself and with its first and second neighbours. However, for simplicity we only show the results for the cell itself for normal and compound Poisson approaches and the results are similar where the cell is combined with its first and second neighbours. Note that we used a significance level 5%. Figure 2 shows the rate of significant clusters for each cell based on the normal and compound Poisson distributions for each scenario.

For scenario 1, where the mean population size is 5000 and (*c *= 5 × 50, *v *= 11 × 50), the normal approach identifies some cells as statistically significant for some simulations. Moreover, when (*c *= 5 × 50, *v *= 12 × 50) and (*c *= 5 × 50, *v *= 14 × 50), the normal approach more closely approximates the compound Poisson results for scenarios 2 and 3, respectively. As expected, the rate of significant cluster in cell 28 is higher than the other cells. Note that in this situation the value of *μ*_*i*_/*σ*_*i *_is relatively small and if k* - 1 is set as the 95th percentile of the normal distribution with mean *μ*_*i *_and variance $\sigma_{i}^{2}$, then the cluster size is too small to have a significant result. That is, the significance level obtained using (5) is just above 0.05 and some cells including cell 28 are not detected as a cluster even though the observed number of events exceeded the cluster size. In this situation, we had to increase the cluster size so that significance could be obtained. This situation demonstrates that with small numbers of events and population the testing algorithm and the normal approach may not be as effective in detecting clusters.

When the mean population size is 10000 and (*c *= 10 × 50, *v *= 22 × 50), the normal approach identifies some cells as significant clusters for some simulations (scenario 4). However, increasing (*c*, *v*) to (10 × 50, 24 × 50), most cells are identified as significant clusters for some simulations (scenario 5), and the normal distribution provides results similar to the compound Poisson in all cells when (*c *= 10 × 50, *v *= 28 × 50) (scenario 6).

Increasing the mean population size to 20000, the normal distribution distinguishes the significant clusters in all cells for (*c *= 20 × 50, *v *= 44 × 50) for some simulations (scenario 7). The rate of significant clusters in the normal and compound Poisson approaches are very close in all cells for (*c *= 20 × 50, *v *= 48 × 50) and (*c *= 20 × 50, *v *= 56 × 50), scenarios 8 and 9.

Note that with increased (*c*, *v*) for each population size and scenario, the rate of significant cells also increases. Moreover, we have observed that the mean proportion of significant cells in scenarios 1 to 9 using the normal method is slightly higher than in the compound Poisson approach (Table 3). The reason is that a continuous random variable (normal distribution) is used to approximate a discrete random variable (compound Poisson distribution) by using the Yates correctness in (5) [15]. In addition, we rounded up decimal cluster sizes to whole numbers to be in line with the spirit of the testing algorithm. Furthermore, the normal approach takes 60, 87, and 182 seconds to complete the simulations for scenarios 3, 6, and 9, respectively. For the compound Poisson approach, 420, 680, and 2100 seconds were required to perform the calculations for these scenarios.

**Table 3:** The mean proportion of significant cells for the compound Poisson (*mp*^*CP*^) and normal (*mp*^*N*^) methods by the nine different scenarios.

Scenario	Mean population size	(*c*, *v*)/50	*mp*^*CP*^	*mp*^*N*^
1	5000	(5,11)	0.043	0.047
2	5000	(5,12)	0.045	0.048
3	5000	(5,14)	0.045	0.049

4	10000	(10,22)	0.046	0.051
5	10000	(10,24)	0.048	0.051
6	10000	(10,28)	0.048	0.053

7	20000	(20,44)	0.052	0.054
8	20000	(20,48)	0.052	0.054
9	20000	(20,56)	0.053	0.055

These simulation results suggest that for each population size, *N*_*i*_, we need to have minimum number of (*c*_*i*_, *v*_*i*_) per cell to have the approximate normal be suitable in these settings. Based on our findings, for mean population sizes 5000, 10000, and 20000, the minimum number of cases/events must average (*c*_*i*_, *v*_*i*_) = (5,14), (10,24), and (20,56), respectively, to have a good approximation to the compound Poisson by the normal approach in these settings.

## Discussion

We proposed an approximate normal to the compound Poisson distribution for the identification of clusters of disease-related events. Incidence or prevalence data are often used for the identification of geographic clusters of disease. In our data example, some individuals returned multiple times to EDs for self-inflicted injuries. Such injuries may be indicative of a health condition and an analysis solely based on the number of individuals with at least one self-inflicted injury may not be as informative as an analysis on the number of self-inflicted injuries. From the ED service perspective, each ED presentation represents a use of services and a loss of information results when individuals are considered rather than services. From the health perspective, multiple presentations may mean that an individual has a more severe health condition or has lesser access to health services than other individuals who present with self-inflicted injuries. Key to the compound Poisson formulation for the determination of the significance level was the specification of the number of events per case. The number of individuals that presented to the ED was assumed to follow a Poisson distribution but the distribution of the number of presentations made by each persons had to be fully specified. The empirical distribution of event frequencies to obtain probability estimates or another distribution can be assumed. Unfortunately, the uncertainty of these estimates and assumptions are not captured in the final testing results and the recursive nature of the compound Poisson probability calculations means that calculations can become prohibitive.

As an alternative to the compound Poisson approach, we have proposed a normal distribution with mean and variance based on the number of events and this formulation does not require the specification of the distribution of the number of events per case. This approach is easily implemented in statistical software and the quantiles are readily calculated. Only a few lines of computer code are required since the normal distribution is a fixture of statistical software whereas statistical software may not have the desired compound Poisson distribution pre-programmed. The incorporation of strata information into the mean and variance of the normal are straight forward. Especially when strata are involved, the normal approach provides a computational advantage. The recursive calculations required by the compound Poisson approach can be problematic when multiple strata variables are included and/or cell population sizes are larger and hence, for larger cluster sizes. In addition users must be aware of numerical instabilities that may arise with the addition of multiple small probabilities. The normal method does not require such recursive calculations and the approximate normal takes at most about 15% of the time. Furthermore, the normal approach's concepts may be more easily conveyed to non-statisticians interested in understanding the methodology associated with cluster detection results. Non-statisticians are more familiar with a normal distribution than a compound Poisson distribution and the approximate normal assumptions are more easily communicated.

We investigated the normal method with our ED data set. For self-inflicted injury presentations both methods identified 12 clusters in common and the normal approach identified one additional cluster. For one tested cell, the cluster size for the compound Poisson approach was 51 and for the normal approach was 50. With 50 observed events, an additional cell had to be combined to have at least 51 events in the compound Poisson approach. No further combination was required for the normal approach and the test yielded a statistically significant result. This discrepancy illustrates the importance of the choice of cluster size. If the cluster size is too small, the observed number of events may exceed the cluster size by a substantive margin and the significance level may not be significant because the cluster size is not far enough in the tail of the distribution. In both methods, the significance level is based on the cluster size and not the observed number of events. If the cluster size is too large, high numbers of events in a particular area may be diluted by the combination of additional areas and statistical significance cannot be obtained. Users can decide what cluster sizes are reasonable for a particular analysis situation, however, such values are often not known before examining the data and it is therefore difficult to provide objective cluster sizes. In particular, this aspect becomes more challenging when the cell population sizes are highly variable. Le et al. [9] provided a testing algorithm to provide objective cluster sizes to be tested in sequence based on the population sizes in individual cells and nearest neighbours. This approach provides minimum cluster sizes necessary to attain statistical significance but increases the number of tests, complicates the presentation of results, leads to *p*-values close to the significance level, and forces the test statistic for significant cells to be linked to the number of cells used in the cluster size determination. We have adapted this approach to the normal method that we proposed since we did not have objective and meaningful cluster sizes known *a priori *for our application.

Using Monte Carlo simulation studies, we further examined the behaviour of the normal approach. We examined cells with mean population sizes of 5000, 10000, and 20000 and fixed the total number of cases, total number of events, and the event distribution. One cell was designated a "true cluster" and had 1.5 times event rate as the other cells. The normal approach works well for most of the simulations situations where the mean number of events is relatively large and when the cell population sizes are relatively large.

## Conclusion

When disease cases can have multiple disease-related events, identifying clusters of event may be important for researchers. These event clusters may signal geographic areas with more severe disease where diseased individuals have more disease-related events than expected by chance. Previously, a compound Poisson distribution was used to obtain the significance level for testing. A drawback to this approach is that the distribution of the number of events per case must be specified. We proposed an approximate normal approach that is characterized by the mean and variance of the number of events and this approximation yielded a simpler calculation of the significance level. We investigated the performance of the approximation using a real data set and in simulation studies. For sufficiently large population sizes, the normal approach provides the same results as the compound Poisson approach.

## Competing interests

The authors declare that they have no competing interests.

## Authors' contributions

MT developed methodology, created computer code, obtained results, and drafted portions of the manuscript. RJR developed methodology, obtained some results, and drafted portions of the manuscript.

## Supplementary Material

Additional file 1 **The result of clustering for each HA with gender and age year as strata based on the compound  Poisson and normal distributions**. This is an excel file. For each HA in the compound Poisson approach, the number of neighbours (*w*_*i*_) to determine the cluster size (*k*_*iw*_) is presented along with the test statistics (*l*_*i*_), the number of visits (*v*_*i*:*l*_) and the population size (*N*_*i*:*l*_) in the *l *+ 1 HAs. Similar definitions are valid for the normal approach. An asterisk (*) denotes significant clusters at the 5% level, unadjusted for multiple testing. The *p*-value for overall clustering is 0. Click here for file
